# Comparison of Penile Cuff Test and Conventional Urodynamic Study Prior to Photoselective Vaporization of Prostate for Benign Prostate Hyperplasia Using a 120 W GreenLight High Performance System Laser

**DOI:** 10.3390/jcm9041189

**Published:** 2020-04-21

**Authors:** Kang Sup Kim, Yong Sun Choi, Woong Jin Bae, Hyuk Jin Cho, Ji Youl Lee, Sung-Hoo Hong, Sae Woong Kim

**Affiliations:** 1 Department of Urology, Incheon St. Mary’s Hospital, College of Medicine, The Catholic University of Korea, Incheon 2143, Korea; prodigy81@catholic.ac.kr; 2 Department of Urology, Eunpyeong St. Mary’s Hospital, College of Medicine, The Catholic University of Korea, Tongil-ro 1021, Eunpyeong-gu 03312, Seoul, Korea; yschoi1008@catholic.ac.kr; 3 Department of Urology, Seoul St. Mary’s Hospital, College of Medicine, The Catholic University of Korea, Seoul 137-701, Korea; Korea.bwoong@catholic.ac.kr (W.J.B.); a0969@catholic.ac.kr (H.J.C.); uroljy@catholic.ac.kr (J.Y.L.); toomey@catholic.ac.kr (S.-H.H.)

**Keywords:** urodynamics, benign prostatic hyperplasia, sensitivity, specificity

## Abstract

Background: We compared the utility of the penile cuff test (PCT) and the conventional urodynamic study (UDS) for the preoperative assessment of patients undergoing scheduled photoselective vaporization of the prostate (PVP) for benign prostate hyperplasia (BPH). Methods: Fifty-nine patients with voiding lower urinary tract symptoms (LUTS) underwent a simultaneous PCT and conventional UDS before PVP. The modified International Continence Society (ICS) nomogram was used to confirm bladder outlet obstruction after measuring maximum urinary flow rate and highest pressure at flow interruption. The PCT and UDS results, in terms of modified ICS nomogram predictions, were compared. Their sensitivities, specificities, and positive and negative predictive values were calculated. Results: Thirty-six patients were diagnosed as obstructed and 23 as non-obstructed/equivocal using the modified ICS nomogram during the PCT. All 36 of the first group were confirmed as obstructed by UDS. Of the 23 diagnosed as non-obstructed/equivocal by the PCT, 14 were confirmed to be non-obstructed by UDS, with nine diagnosed as obstructed. The PCT showed a sensitivity of 80% and a specificity of 100%. The positive and negative predictive values were 100% and 60.9%, respectively. Conclusions: In conclusion, despite our small number of patients, the PCT’s high sensitivity and specificity suggest that it may provide diagnostic information about bladder outlet obstruction before PVP for patients with voiding LUTS. Evidently, the PCT has the potential to be used for some patients as a screening alternative to invasive UDS.

## 1. Introduction 

A urodynamic study (UDS) for bladder pressure measurement is regarded as the gold standard for the clinical evaluation of bladder outlet obstruction (BOO) in patients with benign prostatic hyperplasia (BPH) [1]. However, there is some controversy as to whether UDS needs to be performed prior to surgical treatment [2,3]. Conventional UDS has disadvantages, including its inconvenience, invasiveness, cost, and the risk of urinary tract infection [4]. Furthermore, it requires skilled staff and specialized equipment [5]. For these reasons, in some centers, UDS is not routinely performed for patients with lower urinary tract symptoms (LUTS) and is used only in select cases [5]. Hence, there is substantial interest in determining a noninvasive measurement of bladder pressure during the voiding phase that has high diagnostic accuracy. To overcome the practical disadvantage of invasive UDS, the condom catheter, the intraurethral device, and the inflatable penile cuff have been adopted over the last two decades as less invasive alternatives based on the hypothesis that the external pressure required to interrupt the flow in the urethra or external meatus should be identical to the pressure inside the bladder [6,7,8]. In particular, the penile cuff test (PCT) is an innovative, noninvasive modality for evaluating BOO in patients with LUTS [9]. Some studies have reported good reliability and reproducibility for the PCT [10,11].

Photoselective laser vaporization of the prostate (PVP) has been considered as a valid alternative to transurethral prostatectomy (TURP)—the gold standard surgical modality for treating BPH—due to improvements and developments in laser technology [12]. Several studies have reported that PVP is significantly effective and safe [13,14]. However, there are few reports in the literature about the results of the PCT prior to performing PVP for BOO related to BPH.

Therefore, in this study, we aim to assess and evaluate the validity of the PCT and compare it to invasive UDS in evaluating patients with BOO before performing PVP.

## 2. Patients and Methods

After ethics approval was obtained from the institutional review board in our hospital, all procedures were performed in accordance with the ethical guidelines of the Declaration of Helsinki. This study was approved by the institutional review board of St. Mary’s Hospital, the Catholic University of Korea, Seoul, Korea. The institutional review board number is KC18RESI0778. We reviewed data from patients who underwent a PCT-scheduled PVP at our institution between June 2017 and June 2018. All the patients included in this study underwent a preoperative assessment with transrectal ultrasonography (TRUS), a pressure flow study using the Aquarius^®^ TT UDS system (Laborie Medical Technologies, Toronto, Ontario), and PCT (CT3000, Mediplus Ltd., High Wycombe, UK). This assessment accompanied a general evaluation for LUTS related to BPH, prior to the operation. The assessment consisted of a complete medical history, physical examination (including a digital rectal examination (DRE)), the International Prostate Symptoms Score (IPSS) questionnaire, urinalysis, measurement of serum prostate-specific antigen (PSA), and uroflowmetry (including the maximum urinary flow rate (Qmax) and post-voiding residual urine volume (PVR)). A patient was eligible for inclusion in the study if they met the following criteria: (1) presence of moderate or severe LUTS (IPSS >7); (2) Qmax ≤15 mL/s, PVR ≥100 mL. The exclusion criteria included the following: previous pelvic surgery, urethral stricture, prostate or bladder malignancy, and neurogenic bladder dysfunction. If patients had a high PSA level (>4.0 ng/dL) or DRE or TRUS abnormalities, a TRUS-guided prostate biopsy was performed to exclude malignancy.

Standard UDS was carried out according to the International Continence Society (ICS)’s recommendations [3]. Following preparation of aseptic conditions and local lidocaine gel instillation at the urethra, an 8-Fr double-lumen cystometry catheter and a 6-Fr manometry rectal tube were inserted into the bladder and rectum, respectively. Non-physiological bladder filling was performed using 0.9% saline at a rate of 50 mL per minute. When the patient expressed a strong desire to void, filling was ceased and the patient was asked to micturate into the flowmeter. The intravesical pressure, intra-abdominal pressure, subtracted detrusor pressure, and flow rate were continuously recorded at a sampling frequency of 10 Hz.

The PCT measured bladder pressure in a manner similar to blood pressure measurement using an inflatable cuff. First, a penile cuff was affixed around the penis and in the shaft area. After the patient initiated voiding, the cuff was slowly inflated at a rate of 10 cmH_2_O/s until the urine flow was interrupted. The inflated cuff then rapidly deflated to restart the urine flow and multiple cycles were repeated until micturition was complete. If the cuff pressure increased to >200 cm H_2_O, the test was automatically stopped for safety reasons [9]. The voided volume had to surpass 150 mL for a valid interpretation of the results. The cycle was performed until the conclusion of micturition, usually involving two or more cycles during the void. The measurement of Qmax and an estimate of isovolumetric bladder pressure were obtained and plotted on a modified ICS nomogram to enable categorization into obstructed, not obstructed, or equivocal groups [9,11].

The obtained data were analyzed using SPSS version 20.0 (SPSS Inc., Chicago, IL, USA). The sensitivity, specificity, positive predictive value, negative predictive value, and likelihood ratio were calculated.

## 3. Results

A total of 59 patients were recruited into this study during a 6-month period and provided data suitable for analysis. All patients in the series underwent PVP laser vaporization with preoperative UDS and PCT. Cuff inflation was well tolerated, and no adverse events occurred during or after the PCT. Table 1 shows the patients’ demographic characteristics. The 59 patients had a mean age of 69.6 (range 54–89) years. The mean PSA and prostate volume as evaluated by TRUS were 2.4 ng/dL (range 0.32–10.41) and 52.2 g (range 18–107), respectively. In the UDS, the median Qmax was 7.6 mL/s (range 1.0–14.5), median PVR was 239.1 mL (range 100–494), and the median detrusor pressure at Qmax (Pdet at Qmax) was 69.4 cm H_2_O (range 13–178).

**Figure 1:**
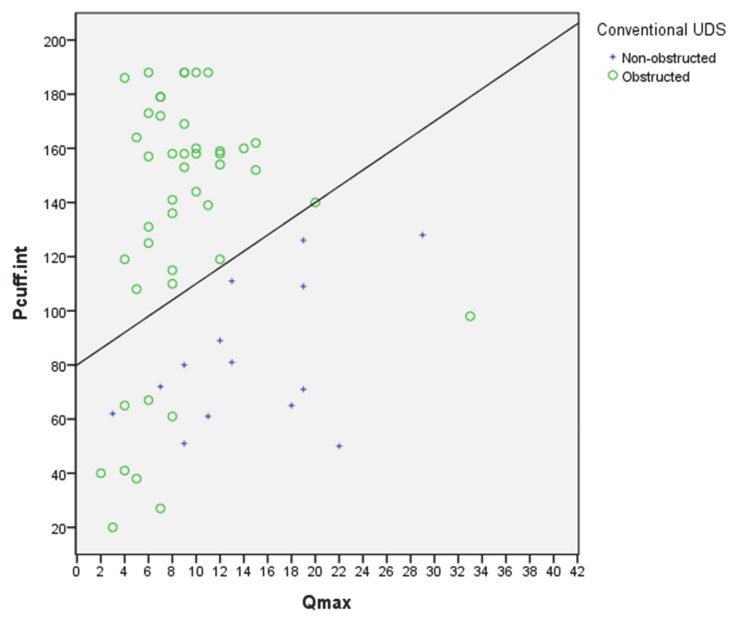
Modified nomogram with data from 59 patients showing the classifications by conventional UDS using the International Continence Society (ICS) nomogram.

Table 2 and Figure 1 show the study results. There were 36 patients classified as obstructed and 23 with no obstruction based on the PCT and the modified ICS nomogram. All patients identified as having obstruction by the PCT were confirmed to have obstruction based on UDS and the ICS nomogram. Furthermore, 45 patients were diagnosed as having an obstruction and 14 patients as being unobstructed by the UDS and the ICS nomogram. Of the 23 patients identified as unobstructed by PCT, nine were classified as obstructed on UDS, and 14 patients were classified as non-obstructed/equivocal on UDS.

The sensitivity, specificity, positive and negative predictive values, and the positive likelihood and negative likelihood ratios for the PCT were calculated and evaluated. The PCT showed a sensitivity of 80% and specificity of 100%, with a positive likelihood ratio of 2.6 (95% CI 2.13–4.02) and a negative likelihood ratio of 0.23 (95% CI 0.1–0.41). The positive and negative predictive values were 100% and 60.9%, respectively. A comparison of the postoperative results of the obstructed group (*n* = 36 patients) and the non-obstructed group (*n* = 14 patients), as classified by the PCT, is shown in Table 3. No significant differences in the preoperative total IPSS, storage IPSS, quality of life (QoL), and PVR were observed between the groups, except for voiding IPSS (*p* = 0.028) and Qmax (*p* = 0.003). After performing PVP, there was no significant difference in the postoperative 1-, 3-, and 6-month total IPSS, storage IPSS, QoL, Qmax, and PVR for both groups. However, the 1-, 3-, and 6-month voiding IPSS was significantly different between the groups (*p* = 0.047, 0.031, 0.033, respectively). Improved total, voiding, and storage IPSS scores, increased Qmax, and a decreased PVR were observed in the two groups compared to the preoperative parameters.

## 4. Discussion

Since our institution began performing minimally invasive prostate surgeries, such as PVP or holmium laser enucleation of the prostate, instead of TURP for treating BOO related to BPH, several years ago [15,16], determining appropriate minimally invasive diagnostic tools for evaluating and discriminating between BOO and impaired detrusor contractility has become mandatory in order to minimize inconvenience and complications caused by conventional UDS. Alternatively, less invasive diagnostic tools, such as ultrasonographic measurements of bladder wall thickness (BWT) or detrusor wall thickness (DWT), external condom catheters, intraurethral devices, and PCT, have been developed and proposed. Several studies have validated that BWT or DWT measurements taken using ultrasound have a higher diagnostic fidelity for identifying BOO than free uroflowmetry, TRUS, or measurements of PVR do [17,18]. Furthermore, the merits of ultrasound measurements of BWT or DWT mean that they are useful for evaluating detrusor overactivity in females or voiding dysfunction in children [19,20]. Measurements of isovolumetric bladder pressure using an external condom catheter are well correlated with the results of UDS for their ability to evaluate BOO accurately [6]. We selected the PCT from among these and performed the present study. The reason we selected this diagnostic tool was that this PCT, commercially known as the Urocuff^TM^, has been validated as being easy to execute, inexpensive, quickly diagnostic, and tends to be selected by 80% of patients instead of UDS [21]. Moreover, the PCT enables discrimination between BOO and possible detrusor underactivity and can assess BOO in patients with types of BOO other than BPH. The primary purpose of this study was to compare the results of PCT and UDS in a diagnostic study of patients with moderate to severe LUTS before performing PVP.

McRae et al. [22] first described PCT in 1995. They placed an inflatable pneumatic cuff around the penile shaft, which is similar to the method used to measure blood pressure. In this study, the cuff was deflated after the initiation of voiding and some patients were sometimes unable to relax the urethral sphincter and began voiding against the obstruction. We adopted the urethral compression–release technique, in which the penile cuff is inflated at a rate of 10 cmH_2_O per second after micturition has started, until urine flow is either intruded or a maximal pressure of 200 cmH_2_O is reached [23]. Several studies have investigated the diagnostic accuracy of the PCT for preoperative evaluation prior to prostate surgery due to BPH. Harding et al. [24] reported the results of a PCT before TURP in a total of 208 patients; they showed that 87% of the patients categorized into the obstruction group and 77% of patients categorized as not obstructed or unobstructed showed improved clinical outcomes after surgery, whereas only 56% of the patients diagnosed as unobstructed experienced good outcomes. These results indicate that PCT is able to improve the prediction of outcomes after TURP. This result was reinforced in a more recent study in which 94% of patients achieved good results after TURP or holmium laser enucleation of the prostate when they were categorized with obstruction using a PCT, versus 70% who were categorized as unobstructed using a PCT [25]. The present study highlights that PCT is a non-invasive and useful diagnostic tool for decision-making prior to the execution of PVP in patients with BOO related to BPH. Our study clarified that the PCT has a sensitivity of 80%, specificity of 100%, positive predictive value of 100%, and negative predictive value of 60.9%. To our knowledge, this study reports the highest positive predictive value (100%) for the PCT when compared to UDS and suggests that the probability of an incorrect diagnosis of obstruction by the PCT is low and that patients could have been provided with definitive data in reported studies on non-invasive pressure testing. Furthermore, our negative predictive value of 60.9% suggests that 39.1% of patients who are incorrectly categorized as unobstructed will still do well. Patients for whom a preoperative PCT categorizes them into the unobstructed category still have a 39.1% opportunity for good clinical results. In particular, our study shows that the IPSS voiding score in patients categorized with obstruction improved to a greater degree than it did in patients categorized into the unobstructed category. It is generally accepted that prostate surgery is more beneficial to patients with BOO presenting with a decreased urinary flow. However, since a decrease in urinary flow can be caused by the weak contraction of the bladder or BOO, all patients with weak urine flow are not automatically categorized as having BOO. It is important to discriminate between BOO and detrusor underactivity before performing prostate surgery, such as TURP and PVP. Thus, the PCT might be a useful diagnostic modality for distinguishing between BOO and detrusor underactivity in patients who are candidates for PVP.

Adverse events such as penile pain or urethral bleeding are rare and self-limiting [24]. Patients accept the PCT well, and the majority of patients favor the PCT over UDS. In addition, both discomfort and distress are lower in the PCT than in UDS [26]. There were no adverse events in our study. In summary, this study demonstrates that a PCT can be utilized in patients with voiding LUTS who will undergo PVP and patients with obstruction categorized by a PCT. Furthermore, PVP improved the IPSS, uroflowmetry, and PVR results.

The limitations of this study include the relatively small number of enrolled patients and the short postoperative follow-up to evaluate PVP’s efficacy in each category of patients. Furthermore, this was a retrospective study and patient enrollment was not randomized. Further studies in a larger number of patients with a longer term postoperative follow-up are mandatory to determine the usefulness of the PCT in the preoperative evaluation of BPH. However, since there are currently few studies that evaluate the PCT prior to PVP for the treatment of LUTS related to BPH, we consider that our study is clinically meaningful.

## 5. Conclusions

Although the retrospective study design and small number of patients are limitations of our study, our results suggest that the PCT has the potential to be used for some patients as a screening alternative to the current conventional UDS with respect to accuracy in predicting BOO without increased morbidity or expense. Furthermore, the PCT is a tolerable procedure and is favored by patients over invasive UDS. The PCT is considered to be an easy and noninvasive measurement technique that is applicable to the diagnosis and treatment planning of patients with LUTS prior to PVP.

## Figures and Tables

**Table 1:** Patients’ preoperative demographic data (59 patients).

Variable	
Age (years), median (IQR)	69.6 (54–89)
PSA (ng/dL), median (IQR)	2.4 (0.32–10.41)
Prostate weight (gm), median (IQR)	52.2 (18–107)
Preoperative total IPSS, median (IQR)	25.8 (8–35)
Preoperative voiding IPSS, median (IQR)	15.7 (5–20)
Preoperative storage IPSS, median (IQR)	10.0 (3–15)
Preoperative QoL, median (IQR)	4.6 (3–5)
UDS	
PdetQmax, median (IQR)	69.4 (13–178)
Qmax (mL/sec), median (IQR)	7.6 (1.0–14.5)
PVR (mL), median (IQR)	239.1 (100–494)
Detrusor underactivity, *n* (%)	3 (5 %)
PCT	
Pcuff.int, median (IQR)	120.7 (40–188)
Qmax (mL/sec)	8.3 (2.0–14.8)
PVR (mL)	173.3 (101–408)

Prostate-specific antigen (PSA); transrectal ultrasonography of the prostate (TRUS); International Prostate Symptom Score(IPSS); quality of life (QoL); urodynamic study (UDS); detrusor pressure at Qmax (PdetQmax); peak urinary flow rate (Qmax); post-voiding residual urine (PVR); penile cuff test (PCT); cuff pressure at interruption (Pcuff.int).

**Table 2:** A comparison of the data from the penile cuff test and conventional urodynamic study (UDS).

	Conventional UDS Obstructed	Conventional UDS Non-Obstructed/Equivocal	Total
Penile cuff test Obstructed	36	0	36
Penile cuff test Non-obstructed/Equivocal	9	14	23
Total	45	14	59

Data represent the number of patients diagnosed as obstructed or non-obstructed for each modality.

**Table 3:** A comparison of follow-up data from cases identified as obstructed or non-obstructed by the penile cuff test.

		Postoperative		
	Preoperative	1 Month	3 Month	6 Month
**IPSS total**				
obstructed	27.6 ± 4.7	9.2 ± 8.9	8.6 ± 9.2	8.4 ± 6.2
non-obstructed	24.8 ± 9.6	8.5 ± 7.1	12.6 ± 9.8	11.9 ± 5.2
*p-*value	0.314	0.153	0.351	0.304
**IPSS voiding**				
obstructed	17.2 ± 2.8	3.9 ± 4.9	3.8 ± 5.9	3.1 ± 4.5
non-obstructed	13.6 ± 5.9	5.7 ± 4.1	7.5 ± 5.7	7.1 ± 2.4
*p-*value	**0.028**	**0.047**	**0.031**	**0.033**
**IPSS storage**				
obstructed	10.2 ± 3.1	5.2 ± 3.8	4.9 ± 3.9	5.3 ± 2.9
non-obstructed	11.2 ± 4.4	2.9 ± 2.7	5.1 ± 5.3	4.8 ± 4.1
*p-*value	0.977	0.353	0.527	0.634
**QoL**				
obstructed	4.5 ± 0.8	2.7 ± 1.6	1.9 ± 1.4	2.2 ± 0.9
non-obstructed	4.6 ± 1.4	3.0 ± 1.4	2.3 ± 1.8	2.1 ± 0.7
*p-*value	0.742	0.625	0.639	0.457
**Q_max_**				
obstructed	6.8 ± 3.1	16.7 ± 8.9	18.9 ± 7.9	19.1± 6.7
non-obstructed	11.1 ± 6.5	20.5 ± 10.0	18.2 ± 9.6	18.2 ± 10.1
*p-*value	**0.003**	**0.27**	**0.862**	**0.754**
**PVR**				
obstructed	63.1 ± 98.4	22.4 ± 29.2	30.8 ± 39.3	34.7 ± 33.5
non-obstructed	102.2 ± 144.6	27.4 ± 32.1	28.1 ± 35.8	32.1 ± 24.5
*p-*value	0.276	0.474	0.882	0.573

International Prostate Symptom Score (IPSS); quality of life (QoL); peak urinary flow rate (Qmax); post-voiding residual urine (PVR); the *p* value compares the obstructed and the non-obstructed values.
